# Mental Diseases

**Published:** 1883-04-20

**Authors:** 


BEAUTIFUL POCKET DRUG CASE.—Among the most unique and beautiful Pocket Drug Cases we have ever seen was one selected for the successful contestant for the prize in Physiology at the late commencement of the Southern Medical College. It was from the splendid drug house of McKesson & Robbins, New York. The vials were all filled with beautiful gelatine coated pills and granules. The size of the case, the plan of opening, the number of vials, the assortment of drugs, and the entire get up of the case, was the very acme of beauty, usefulness and convenience.

Established September 1, 1881. The Philadelphia Hospital for Skin Diseases, No. 923 Locust street, Philadelphia, Pennsylvania, is under the control of the following Board of Managers:

| | |
|---|---|
| Robert E. Rogers, M. D., | Hon. A. K. McClure, |
| William H. Pancoast, M. D., | William S. Jenney, M. D., |
| Rev. George F. Wiswell. D. D., | William B. Atkinson, M. D., |
| C. W. McKeehan, Esq., | Lawrence Wolff, M. D., |
| George W. Fairman, Esq., | George H. Brown, Esq. |

### *MENTAL DISEASES.*

The study of Mental diseases is attracting more attention than in former years. The nature and operations of the human mind and its relations to the physical part of the man, and the extent to which it is dependent upon the brain for its action, are subjects of deep and important interest. The advocates of Scientific Materialism, as Heckel, Ingersoll, and others, have grown bold and confident in rejecting all necessity for the seperate or independent existence of a soul or thinking principle, claiming that thought is but the product of brain secretion, or a result of molecular motion of brain substance, and that with the death or destruction of the brain, all mental operation on the part of the individual ceases, and he goes into absolute nonentity. The doctrine of spontaneous generation, the evolution of forms and species, and the survival of the fittest, is sufficient with this class of scientists to ccount for the existence of man and of all animals, as we now find them upon the earth, and there is no God and no necessity for a God in the universe. While a few hold to these doctrines, alarming the Christian world with the boldness of their assertions, and the extent and ability of their literature which finds access to nearly all classes of periodicals and magazines in our country, yet those who are calmly surveying the field in a spirit free from prejudice and infidel proclivity, see no great danger of the subversion of the old and scriptural doctrine of the separate existence of the mind and soul of man as a real entity, which can and does occasionally act independently of the body, even in this life, and which, at death, comes out of the body as the caterpillar from its chrysalis, a real, substantial and immortal existence.

The committee of the National Association for the Protection of the Insane and the prevention of Insanity, realizing the increasing importance of the study of mental diseases, suggest the establishing of special chairs in all our schools for instruction in Mental Science and Mental diseases. The increased prevalence of insanity, and the growing importance of a better knowledge of mental phenomena, make the suggestion a timely and worthy one, and we doubt not it will meet with the ready acquiescence of the medical profession throughout our land.

